# A novel transmission-based test of association for multivariate phenotypes: an application to systolic and diastolic blood pressure levels

**DOI:** 10.1186/1753-6561-8-S1-S71

**Published:** 2014-06-17

**Authors:** Tanushree Haldar, Indranil Mukhopadhyay, Saurabh Ghosh

**Affiliations:** 1 Human Genetics Unit, Indian Statistical Institute, 203, B.T. Road, Kolkata 700108, India

## Abstract

Unlike case-control studies, family-based tests for association are protected against population stratification. Complex genetic traits are often governed by quantitative precursors and it has been argued that it may be a more powerful strategy to analyze these quantitative precursors instead of the clinical end point trait. Although methods have been developed for family-based association tests for single quantitative traits, it is of interest to develop such methods for multivariate phenotypes. We propose a novel transmission-based approach based on a trio design using a simple logistic regression to test for association with a multivariate phenotype. We use our proposed method to analyze data on systolic and diastolic blood pressure levels provided in Genetic Analysis Workshop 18. However, we find that the bivariate analysis of the two phenotypes did not provide more promising results compared to univariate analyses, suggesting a possibility of a different set of major genetic variants modulating the two phenotypes.

## Background

The family-based design [1] for detecting association is a popular alternative to population-based case-control studies since it circumvents the problem of population stratification. Moreover, in spite of successful identification of a large number of common variants associated in various complex traits, the proportion of total variation in a trait explained by these variants has been minimal and has motivated a search for rare variants that could explain the "missing heritability". Because rare variants are likely to be more frequent in large families compared to the general population, it may be a more prudent strategy to test for transmission disequilibrium in pedigrees to identify these variants. Although transmission-based tests for association of both binary and quantitative traits have been extensively studied [1-4], extension of such tests for multivariate phenotypes is of current research interest. We have developed a computationally simple logistic regression-based test that models the probability of transmission of the minor allele at a single-nucleotide polymorphism (SNP) from a heterozygous parent conditioned on the multivariate phenotype values of the offspring. We apply our proposed method to analyze systolic and diastolic blood pressure levels in a pedigree using longitudinal data over four time points provided in Genetic Analysis Workshop 18 (GAW18).

### Data description

For our analyses, we use pedigree data on systolic blood pressure (SBP) levels and diastolic blood pressure (DBP) levels at four different time points for 453 individuals along with their genotypes at all of the available 456,752 variant sites distributed over 11 autosomal chromosomes. In addition to age, we used smoking status and medication indicator (both defined as binary variables) at each time point of examination as covariates, as these factors could be potential confounders in the association analyses. Both the SBPand the DBP levels were adjusted for these covariates for each time point and the tests for transmission disequilibrium were performed on the adjusted phenotypes.

## Methods

### Statistical methodology

#### Imputation of missing phenotype values and covariate adjustment

Data on the two phenotypes and the covariates are not available for all individuals at every time point. The assumption of multivariate normality provides a computationally elegant framework for the expectation maximization (EM) algorithm [5] to estimate parameters when data are missing. Blood pressure levels have traditionally been believed to follow a lognormal distribution. Although the Kolmogorov-Smirnov test did not show any significant departure from normality for the SBP and DBP levels at any of the time points, some of the *p-*values are very close to the threshold of 0.05. We thus perform a logarithmic transformation on each of the phenotypes to induce normality. We use an unrelated set of 142 individuals from the pedigrees for whom data on all the variables are available to estimate the missing log-transformed phenotype values using data on the available phenotype values. Suppose the vector of log-transformed values of any of the two phenotypes at the four time points is represented as ***X ***= (*X*_1_, *X*_2_, *X*_3_, *X*_4_). If ***Y ***denotes the vector comprising those components of ***X ***that are missing and ***Z***is the components that are available for an individual, ***Y ***is estimated via an EM algorithm as the expectation of ***Y ***conditioned on ***Z ***and is given by μ_Y _Σ_*YZ *_Σ_*ZZ*_^−1^(***Z***-μ_Z_), where, μ_Y _and μ_Z _are the mean vectors of ***Y ***and ***Z***, respectively; Σ_*YZ *_is the matrix of covariance between ***Y ***and ***Z***, while Σ_*ZZ *_is the dispersion matrix of ***Z***. We perform a linear regression of the log-transformed values of each of the two phenotypes (available as well as imputed) at each time point on age, smoking status, and medication indicator. We plug-in the parameter estimates of the mean vector and variance-covariance matrix of the log-transformed phenotypes obtained via the EM algorithm to estimate the missing log-transformed values of each phenotype conditioned on the available log-transformed values of that phenotype at every time point for the remaining individuals in the pedigree. We then use the regression equation at each time point to obtain the residuals for all individuals in the pedigree for whom data are available on all the covariates.

#### Test for transmission disequilibrium using logistic regression

The phenotypes for our association analyses are the adjusted SBP and DBP levels at each time point obtained using the algorithm described in the preceding section. We use a novel binary logistic regression framework to test for association of a SNP with a multivariate phenotype. For each SNP, we consider all trios in the pedigree with at least 1 heterozygous parent at that SNP, selecting one sib at random from each sibship. Suppose ***X ***= (*X*_1_, *X*_2_, *X*_3_, ...,*X*_k_) denotes a vector of *k *phenotypes and *W*is anindicator random variable (1 or 0) denoting whether a heterozygous parent at a SNP transmits the minor allele or not. We model the conditional distribution of *W *given ***X ***using a logistic link function given by:

$$P\left(W=1|X_{1},X_{2},...,X_{k}\right)=\frac{\text{exp}\left\{\beta_{0}+\sum_{i=1}^{k}\beta_{i}\left(X_{i}-\mu_{i}\right)\right\}}{1+\text{exp}\left\{\beta_{0}+\sum_{i=1}^{k}\beta_{i}\left(X_{i}-\mu_{i}\right)\right\}}$$

where, μ_i_is the mean of *X_i _*in the population thatis estimated by the sample mean and the parameters $\beta_{0},\beta_{1},\beta_{2},...,\beta_{k}$ are estimated using the method of maximum likelihood.

We note that even though this model is in similar lines as Waldman [6], it captures the pattern of transmission disequilibrium in a more optimal fashion as the phenotypes are corrected for their means, making this model more powerful. The test for transmission disequilibrium is equivalent to testing *H_0_*: *β_1_*=*β_2 _*= ... =*β_k_*= 0 versus *H*_1_: not *H*_0 _and the log-likelihood ratio test statistic is distributed as chi-squares with *k *degrees of freedom under the null hypothesis. We compare the relative performances of 3phenotype vectors in detecting association: (a) *T*_1_: the adjusted SBP levels summarized by the first two principal components across the four time points; (b) *T*_2_: the adjusted DBP levels summarized by the first two principal components across the four time points; and (c) *T*_3_: a bivariate phenotype comprising the adjusted SBPand the adjusted DBP levels summarized by the first two principal components corresponding to each of the phenotypes across the four time points. The above choice of principal components is motivated by the fact that 75% of the variation in each of the two phenotypes is explained by the corresponding first two principal components. To correct for multiple testing, we use the false discovery rate procedure [7] with an overall rate of 0.05.

## Results

The pedigree is made up of 95 distinct pairs of parents. Thus, our transmission disequilibrium analyses are based on 95 independent trios. Given that most parents have multiple offspring, there exists a large number of possible sets of trios if 1 sib is selected at random from each sibship made up of two or more sibs. We consider 1000 such possible sets of trios at random. Because transmissions only from heterozygous parents are relevant for the proposed test for transmission disequilibrium, we analyze only those SNPs that are made up of at least 25 informative trios for efficient estimation of parameters in the logistic regression. We also exclude those SNPs that show significant deviation from the Hardy-Weinberg equilibrium based on the unrelated set of 139 individuals for whom genotype data are available, and use Bonferroni correction for multiple testing.

The tests for association based on the proposed logistic regression are carried out on 426,193 SNPs. Among the phenotype vectors considered, contrary to our expectation that *T*_3 _(the phenotype made up of the first two principal components of both SBP and DBP levels) would be more powerful in detecting association, *T*_1 _(the phenotype made up ofthe first two principal components of SBP levels) provides the most promising association finding. The SNPs *rs4754220 *and* rs12419678* on chromosome 11 attains genome-wide significance (based on the desired false discovery rate of 0.05) with *T*_1 _in 37 and 35 of the 1000 sets of trios, respectively. On the other hand, the SNP *rs13301156 *on chromosome 9 exhibits significant evidence of transmission disequilibrium with *T*_2 _(first two principal components of DBP) in 24 sets of trios. These three SNPs also rank among the top five SNPs significantly associated with *T*_3_, although in less than 10 sets of trios.

## Conclusions

We have developed a simple binary logistic regression model that incorporates multiple phenotypes for transmission-based association analyses of the multivariate phenotype vector. The method does not involve any modeling of the correlation structure within the components of the multivariate phenotype as required in likelihood-based approaches and, consequently, is more robust with respect to distributional assumptions. On the other hand, the method does not reduce the multivariate phenotype vector to principal components, thus circumventing the problem of biological interpretations of derived phenotypes.

The SNPs *rs4754220 *and *rs12419678 *that exhibited the most significant evidence of linkage disequilibrium with SBP values are located in the intronic region of the gene *CWF19L2* (CWF19-like 2, cell-cycle control) on 11q22.3. Studies show that RNA expression of this gene is upregulated in humans for inflammatory cardiomyopathy [8]. On the other hand, the SNP *rs13301156 *that yields significant evidence of association with DBP levels is located in the intergenic region between the genes *RPS6P13 *(ribosomal protein S6 pseudogene13) and *GAS1 *(growth arrest-specific 1) on 9q21.3. The RNA expression of *RPS6P13 *has been reported to bedownregulated in humans for coronary collateralization [9], while the RNA expression in *GAS1 *has been reported to be upregulated for arrhythmogenic right ventricular cardiomyopathy in humans [10].

It is expected that if a genetic variant modulates multiple phenotypes, a multivariate analysis will be more powerful than separate univariate analyses in detecting association with the genetic variant. However, we find that the association test for the bivariate phenotype is less powerful than the tests for SBP levels and DBP levels separately. Moreover, the most significant association findings obtained for the bivariate phenotype form a disjoint union of those obtained for the two phenotypes separately. Consequently, it is possible that althoughthere may be common genes modulating both SBP and DBP levels, the major genetic variants for the two phenotypes may be different and the bivariate phenotype contains minimal additional information on the variants compared to any of the two phenotypes.

The proposed transmission-based association test can incorporate multiple sibs within a sibship by considering the transmission to each sib separately. However, such a test is strictly a valid test only for linkage. Althoughthe presence of association increases the power to detect transmission disequilibrium, the rejection of the null hypothesis does not necessarily imply the presence of linkage disequilibrium. When we perform our proposed test with all sibs within each sibship, we obtain large clusters of significant SNPs since linkage exists over much larger distances on the genome compared to linkage disequilibrium. However, we find that the clusters on chromosomes 9 and 11 include the three SNPs that provided the most significant evidence of association. We are currently exploring the theoretical properties of various methods to integrate the test statistics (such as the mean or the maximum order statistic) for the different sets of trios (considering 1sib at random from each sibship) into a combined test statistic.

## Competing interests

The authors declare that they have no competing interests.

## Authors' contributions

SG and TH developed the proposed method. AMand TH wrote the computer codes and performed the data analyses. TH participated in the compilation and interpretation of the results. SG drafted the manuscript. All authors read and approved the final manuscript.
